# Training Savvy Caregiver Program Group Leaders Through an Online Course

**DOI:** 10.1093/geroni/igab046.1512

**Published:** 2021-12-17

**Authors:** Kenneth Hepburn, Carey Sherman, John Hobday, Lai Reed

**Affiliations:** 1 Emory University, Atlanta, Georgia, United States; 2 Family Care Strategies, LLC, Ann Arbor, Michigan, United States; 3 Healthcare Interactive, inc, St. Louis Park, Minnesota, United States

## Abstract

A significant factor limiting organizations’ implementation of the Savvy Caregiver program, a widely disseminated dementia caregiver psychoeducation course, is the need to provide training to program leaders to ensure their understanding of Savvy core principles and strengthen their teaching and coaching skills. Such training has typically been provided through in-person group sessions led by the Savvy developers. To facilitate broader availability, we have embarked on an NIA-supported program to develop a fully online self-paced Savvy train-the-trainer course. The course, delivered individually on a widely used teaching platform, is in seven sections: the first introduces Savvy principles and the trainer role; the next six cover the content and teaching strategies of each of Savvy’s six sessions. In the first development phase, 33 individuals from 13 organizations across the country took part in training (average age 49.5; almost all college level or professional women). Qualitative interviews with 11 trainees and debriefing sessions with others yielded consistently positive responses: the training enhanced their own appreciation for caregiving; they endorsed the self-paced learning and; and it established expectations for positive benefits of Savvy for caregivers. Trainees’ feedback has led to several improvements, including resolving reported technical glitches (e.g., navigating the course). New videos illustrating group delivery methods have been added. Fidelity monitoring strategies are supported as organizations have been encouraged to augment the online training by convening meetings of trainees while in training to enable role playing, and greater personalization is achieved via post-training Zoom meetings with trainees and the Savvy training team staff.

